# Identifying gene-gene interactions that are highly associated with Body Mass Index using Quantitative Multifactor Dimensionality Reduction (QMDR)

**DOI:** 10.1186/s13040-015-0074-0

**Published:** 2015-12-14

**Authors:** Rishika De, Shefali S. Verma, Fotios Drenos, Emily R. Holzinger, Michael V. Holmes, Molly A. Hall, David R. Crosslin, David S. Carrell, Hakon Hakonarson, Gail Jarvik, Eric Larson, Jennifer A. Pacheco, Laura J. Rasmussen-Torvik, Carrie B. Moore, Folkert W. Asselbergs, Jason H. Moore, Marylyn D. Ritchie, Brendan J. Keating, Diane Gilbert-Diamond

**Affiliations:** 1 Computational Genetics Laboratory, Department of Genetics, Geisel School of Medicine at Dartmouth, Dartmouth-Hitchcock Medical Center, 706 Rubin Building, HB7937, One Medical Center Dr, Lebanon, NH 03756 USA; 2 Center for Systems Genomics, Department of Biochemistry and Molecular Biology, 512 Wartik Laboratory, The Pennsylvania State University, University Park, PA 16802 USA; 3 Centre for Cardiovascular Genetics, Institute of Cardiovascular Science, Faculty of Population Health Sciences, University College London, 5 University Street, London, WC1E 6JF UK; 4 Division of Transplant Surgery, Perelman School of Medicine, University of Pennsylvania, 3400 Spruce Street, 2 Dulles Pvln, Philadelphia, PA 19104 USA; 5 Department of Genome Sciences, University of Washington, 3720 15th Ave NE, Seattle, WA 98195-5065 USA; 6 Group Health Research Institute, Metropolitan Park East, 1730 Minor Avenue, Suite 1600, Seattle, WA 98101-1448 USA; 7 The Joseph Stokes Jr. Research Institute, The Children’s Hospital of Philadelphia, Office 1016 Abramson Building, Room 1216E, 3615 Civic Center Blvd, Philadelphia, PA 19104 USA; 8 Division of Medical Genetics, Department of Medicine, University of Washington, Health Sciences Building, K-253B, Medical Genetics, Box 357720, Seattle, WA 98195-7720 USA; 9 Center for Genetic Medicine, Northwestern University Feinberg School of Medicine, 303 E. Superior Street, Lurie 7-125, Chicago, IL 60611 USA; 10 Department of Preventive Medicine, Northwestern University, Feinberg School of Medicine, 680 N Lake Shore Drive, Suite 1400, Chicago, IL 60611 USA; 11 Center for Human Genetics Research, Vanderbilt University School of Medicine, 519 Light Hall, Nashville, TN 37232 USA; 12 Department of Cardiology, Division Heart and Lungs, University Medical Center Utrecht, Room E03.511, P.O. Box 85500, 3508 GA Utrecht, The Netherlands; 13 Institute of Cardiovascular Science, University College London, London, UK; 14 Durrer Center for Cardiogenetic Research, ICIN-Netherlands Heart Institute, Utrecht, The Netherlands; 15 Institute for Biomedical Informatics, The Perelman School of Medicine, University of Pennsylvania, 1418 Blockley Hall, 423 Guardian Drive, Philadelphia, PA 19104-6021 USA; 16 University Medical Center Utrecht, Utrecht, The Netherlands; 17 Institute for Quantitative Biomedical Sciences at Dartmouth, Hanover, NH USA; 18 Department of Epidemiology, Geisel School of Medicine at Dartmouth, One Medical Center Drive, 7927 Rubin Building, Lebanon, NH 03756 USA

**Keywords:** Obesity, Epistasis, Gene-gene interaction, Multifactor dimensionality reduction, GWAS

## Abstract

**Background:**

Despite heritability estimates of 40–70 % for obesity, less than 2 % of its variation is explained by Body Mass Index (BMI) associated loci that have been identified so far. *Epistasis*, or gene-gene interactions are a plausible source to explain portions of the missing heritability of BMI.

**Methods:**

Using genotypic data from 18,686 individuals across five study cohorts – ARIC, CARDIA, FHS, CHS, MESA – we filtered SNPs (Single Nucleotide Polymorphisms) using two parallel approaches. SNPs were filtered either on the strength of their main effects of association with BMI, or on the number of knowledge sources supporting a specific SNP-SNP interaction in the context of BMI. Filtered SNPs were specifically analyzed for interactions that are highly associated with BMI using QMDR (Quantitative Multifactor Dimensionality Reduction). QMDR is a nonparametric, genetic model-free method that detects non-linear interactions associated with a quantitative trait.

**Results:**

We identified seven novel, epistatic models with a Bonferroni corrected *p*-value of association < 0.1. Prior experimental evidence helps explain the plausible biological interactions highlighted within our results and their relationship with obesity. We identified interactions between genes involved in mitochondrial dysfunction (*POLG2*), cholesterol metabolism (*SOAT2*), lipid metabolism (*CYP11B2*), cell adhesion (*EZR*), cell proliferation (*MAP2K5),* and insulin resistance (*IGF1R*). Moreover, we found an 8.8 % increase in the variance in BMI explained by these seven SNP-SNP interactions, beyond what is explained by the main effects of an index *FTO* SNP and the SNPs within these interactions. We also replicated one of these interactions and 58 proxy SNP-SNP models representing it in an independent dataset from the eMERGE study.

**Conclusion:**

This study highlights a novel approach for discovering gene-gene interactions by combining methods such as QMDR with traditional statistics.

**Electronic supplementary material:**

The online version of this article (doi:10.1186/s13040-015-0074-0) contains supplementary material, which is available to authorized users.

## Background

Obesity is a major risk factor for various diseases such as - heart disease, type 2 diabetes and even certain types of cancer [1, 2]. Approximately, one-third of the adult population in the U.S. is categorized to be obese [3]. Globally, obesity has the potential to affect 1.12 billion individuals by 2030 [4]. In the U.S. alone, the economic burden associated with obesity has been estimated to be around $147 billion/year in healthcare costs and loss of productivity of affected individuals [5]. Moreover, obesity no longer affects only industrialized nations, but it is also making its mark in developing nations, especially among children [3, 6].

Although the current epidemic proportions of obesity can be largely attributed to our lifestyle and food choices, there is also a strong genetic component of obesity. Twin and adoption studies have provided heritability estimates of 40–70 % for obesity [7, 8]. Such studies have also found that obesity tends to cluster within families, and that monozygotic twins show greater concordance in Body Mass Index (BMI) and adiposity metrics versus dizygotic twins. Technological advancements in genomics and highly characterized genome-wide reference maps in major populations allow researchers to query a million or more genetic variants by designing genome-wide association studies (GWAS), [9–11] and so far, researchers have identified BMI-related signals in 32 loci that are associated with the trait at a genome-wide level [1]. However, these primary associations have been able to explain only about 2 % of the variation observed in BMI [1].

The limited success of GWAS has often been attributed to the linear framework employed by these studies. Although, single locus analysis strategies have had success in certain diseases such as age-related macular degeneration and breast cancer [12–15], many complex diseases are likely the result of interactions between genetic loci – *epistasis* [9, 11, 16]. The ubiquitous nature of epistasis has been discussed previously, and it has highlighted the importance of designing our studies to embrace the genomic and environmental context of Single Nucleotide Polymorphisms (SNPs), by specifically searching for non-linear interactions between genetic loci [17, 18].

In this study we aimed to identify interactions between SNPs that are associated with BMI using data from 18,686 individuals across five highly characterized National Heart, Lung and Blood Institute (NHLBI) study cohorts. Individuals were genotyped using the gene-centric ITMAT-Broad-CARe (IBC) array containing approximately 50,000 SNPs.

## Methods

### Participants

**Fig. 1:**
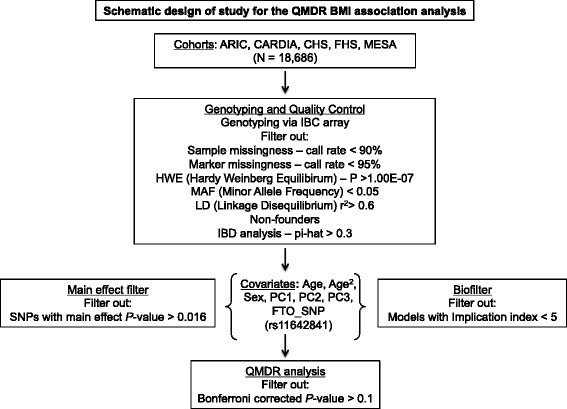
Schematic design of the QMDR (Quantitative Multifactor Dimensionality Reduction) analysis for identifying SNP-SNP interaction models associated with BMI. Genotyping was performed using the IBC (ITMAT-Broad-CARe) array. The workflow also includes the initial quality control procedures, subsequent association analyses, and covariate adjustment steps performed

Figure 1 illustrates the overall study design. Genotype and phenotype information were initially combined for a total of 18,686 individuals of European descent from the following studies: Atherosclerosis Risk in Communities (ARIC) [19]; Coronary Artery Risk Development in Young Adults (CARDIA) [20]; Cardiovascular Health Study (CHS) [21]; Framingham Heart Study (FHS) [22]; and Multi-Ethnic Study of Atherosclerosis (MESA) [23] (Additional file 1: Table S1).

### Genotyping and quality control

Genotyping was performed using the gene-centric ITMAT-Broad-CARe (IBC) array. This array was designed specifically to test over 2,000 loci implicated in various cardiovascular, metabolic and inflammatory phenotypes [24]. The array contains 47,451 SNPs. Samples with a call rate less than 90 % were excluded. Additionally, SNPs with a call rate less than 95 %, with an exact test of Hardy-Weinberg equilibrium *p*-value greater than 1.00E–07 or a minor allele frequency (MAF) < 0.05 were also excluded. SNPs were further tested for linkage disequilibrium (LD) – a SNP was removed from each pair of SNPs that had an LD (r^2^) ≥ 0.6. This reduced our dataset to 17,268 individuals and 28,453 SNPs. Non-founder individuals were also removed from the study population. To check for relatedness between individuals, markers were used for an Identity-by-descent (IBD) analysis using PLINK [25]. For pairs of individuals with a pi-hat ($\widehat{\pi}$) value greater than 0.3, one individual was removed. Complete phenotype data was also required for inclusion of an individual in the analysis. This resulted in a final dataset of 15,737 individuals and 28,453 SNPs. To decrease both the computation time and the multiple testing burden two filtering strategies were employed [26]. These are described in more detail below.

### Marker selection

#### Main effect filter

As an additional filtering step, SNPs were tested for their independent association with the continuous BMI outcome using linear regression. Upon visual inspection of the distribution of *P*-values, a cut-off value of *P* < 0.016 was chosen, as there was a distinct separation between SNPs exhibiting a stronger main effect and the rest of the SNPs at this cut-off. This resulted in a final list of 498 markers for further analysis [27].

### Biofilter

As a parallel filtering procedure, SNPs were also analyzed using Biofilter [28]. Biofilter is a knowledge-based approach that enables the analysis of multi-SNP interactions in a large dataset. The software identifies multi-SNP models that exhibit marginal effects on a phenotype, but are also biologically plausible. It combines information from multiple public knowledge sources such as Gene Ontology (GO), Kyoto Encyclopedia of Genes and Genomes (KEGG), Database of Interacting Proteins (DIP) and the Protein Families Database (Pfam) [29]. These sources provide information regarding pairs of genes that may be putative sources of epistasis and relate genes to one another through their mutual participation in biological processes, signaling pathways, protein-protein interactions as well as via the structural similarity between protein motifs. Biofilter measures the strength of the knowledge-based support for a given multi-SNP model with an *implication index*. The implication index is the sum of the number of supporting data sources for each of the genes in a given gene-gene relationship. For our analysis, models with an implication index of five or greater were retained, resulting in a list of 1815 markers (22,644 SNP-SNP models). The selected implication index cut-off was slightly more stringent than those used in previous studies [30].

### Statistical analyses

#### Covariate adjustment

Baseline BMI values were regressed on age, age^2^, sex, the first three principal components of race computed using EIGENSTRAT software [31] and the index SNP rs11642841 in the *FTO* region. SNPs in the *FTO* locus are some of the strongest genetic associations identified for obesity risk [32]. Hence, adjustments were made for a SNP in the *FTO* locus to increase our ability to identify SNP-SNP models that were not primarily driven by the strong main effect of this gene. The residual BMIs from this regression model were then used as the continuous outcome variable in the QMDR analysis.

### Association analysis – QMDR

SNPs obtained from the two parallel filtering procedures described above, were tested for association with the continuous BMI outcome using Quantitative Multifactor Dimensionality Reduction (QMDR) [33]. QMDR is an extension of the two-class MDR algorithm that can detect and characterize epistatic SNP-SNP interactions in the context of a quantitative trait [34].

The original MDR algorithm was designed as a data reduction approach to identify multi-locus genotype combinations that are associated with high or low risk of disease [34]. Within a given dataset of *m* SNPs, *k* SNPs can be selected to examine a *k*-order interaction. MDR then constructs a contingency table for these *k* SNPs, and calculates case–control ratios for each of the possible multi-locus genotypes. Next, the case–control ratio for each multi-locus genotype is compared to the global case–control ratio for the whole dataset. Accordingly, a genotype is considered *high-risk* if its case–control ratio exceeds the global case–control ratio. Alternatively, it is considered to be *low-risk*.

However, when QMDR constructs a similar contingency table for *k* SNPs, it compares the mean value of the phenotype to the overall mean of the phenotypic trait within the dataset. Hence, a genotype combination is considered *high-level* if its mean value is larger than the overall mean of the phenotypic trait within the dataset. Otherwise, it is considered *low-level*. Next, QMDR combines the *‘high-level’* and *‘low-level’* genotypes into respective groups, and compares the phenotypic outcomes between these two groups using a *T*-test.

QMDR also uses a 10-fold cross-validation procedure similar to the original MDR algorithm. The dataset is divided into 10 portions – 9 portions are used as a training dataset, and the remaining portion is used as a testing dataset. Next, the training t-statistic is calculated for each *k*-way interaction in the training dataset. The *k*-way model with the best training score is then used to predict the case–control status in the testing dataset. Ultimately, the best *k*-order interaction model is chosen based upon the training t-statistic and the highest testing t-statistic is used to select the best overall model for the dataset.

In the current analyses, we utilized QMDR to specifically test filtered SNPs for all possible two-way (SNP-SNP) interaction models that are associated with the continuous BMI outcome based on their training T-statistic scores. Amongst these models, we selected the 100 best overall SNP-SNP models based on their respective testing T-statistic scores.

### Permutation testing to assess statistical significance

Permutation procedures were performed to determine a cut-off threshold for an α = 0.05 significance level. A 1,000 permutations were performed, and in each permuted dataset the 100 best two-way SNP models were selected based on their T-statistic training and testing values. The null distribution of the 100 best SNP models and T-statistic values obtained from all permutations was utilized to calculate *P*-values for SNP-SNP models. *P*-values were also corrected for multiple testing using standard Bonferroni corrections.

### Assessing the non-additive nature of identified pairwise interactions

A 1,000 permuted datasets were created using the *explicit test of epistasis*, by shuffling genotypic data for each SNP [35]. However, genotype frequencies were maintained so that independent main effects were preserved while non-linear interactions were randomized. Linear regression was used to model the identified statistically significant SNP-SNP interactions in relation to BMI within the original and permuted datasets. Interactions between SNPs were coded as Cartesian products within the regression model. The nine possible two-locus genotypes were coded from 0–8 (Additional file 2: Figure S1). The null distribution was created using the F-statistic values for the regression models from the 1000 permuted datasets. This was used to calculate the ‘explicit epistasis’ *P*-value associated with the original pairwise interactions that were identified.

### Assessing the added variance in BMI explained by identified pairwise interactions

Linear regression models were used to assess the added variance of the quantitative BMI trait explained by the statistically significant SNP-SNP interactions identified in our analyses. The reduced regression model was built by including the main effect of the index SNP rs11642841 in the *FTO* region, and the main effects of all SNPs within our identified interactions. The full regression model included the identified pairwise interactions in addition to the terms from the reduced model. Adjusted R^2^ values were used to assess the variance explained by both models. Additionally, a likelihood ratio test was used to compare both models.

### Biological evidence for identified pairwise interactions

To identify known biological evidence supporting the statistically significant pairwise interactions, we mapped each SNP to a corresponding gene using information from dbSNP (build 139) and SCANdb (http://www.scandb.org). We also searched for evidence of functional relationships between interacting genes using the Integrated Multi-Species Prediction (IMP) web server [36]. IMP integrates information from a large number of sources including experimentally verified data from gene expression studies, IntAct, MINT, MIPS, and BioGRID databases in order to provide a predictive probability that two genes work together within a given biological process.

### Replication analyses

SNP-SNP models that reached a Bonferroni-corrected *P*-value < 0.1 were selected for replication in the eMERGE I-660 dataset [37]. This dataset was imputed using data from the 1000 Genomes Project. Detailed information regarding the replication dataset is presented in Additional file 3: Table S2. SNPs that are in high LD with the SNPs within these interactions were identified using SNAP [38]. These SNPs were then used to generate a list of ‘proxy’ SNP-SNP models that represented the original interaction models. Both the original and proxy SNP-SNP models were tested for replication in the independent dataset. The same QMDR analysis procedure described earlier was used to specifically test for these models in the eMERGE dataset. Additional file 4: Table S3 shows the number of LD expanded models that were generated and tested for each of the original SNP-SNP interactions.

## Results

### Main effect filter

**Fig. 2:**
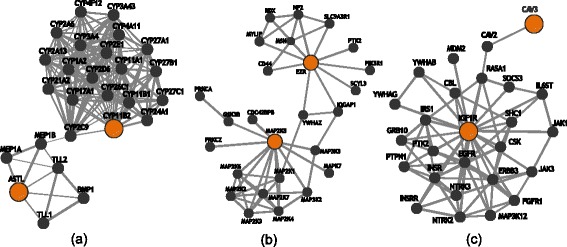
Functional relationship networks generated from Integrated Multi-Species Prediction (IMP) from identified SNP- SNP interactions that are highly associated with BMI. Identified SNPs were mapped to their respective genes. Gene pairs were used to query IMP to make functional connections between them. IMP is a web-based tool that mines empirical data to provide a predictive probability that two genes have a functional relationship. Nodes in the network represent genes. Query genes are represented with larger nodes. Edges between nodes represent a functional relationship between two genes. Shown are interactions between (**a**) rs749457 in ASTL and rs1799998 in CYP11B2 (**b**) rs3102976 in EZR and rs997295 in MAP2K5 (**c**) rs2268484 in CAV3 and rs8038415 in IGF1R

Using the set of SNPs that emerged from the main effect filter, QMDR analysis identified seven novel SNP-SNP interaction models that were associated with BMI (Bonferroni corrected *P-*value <0.1) (Table 1). These SNP-SNP models also reflect strong epistatic relationships. *P*-values associated with the non-additive nature of these interactions are also presented in Table 1. We also queried the biological and functional context of these interactions using IMP. However, since both *FLJ30838* and *C7orf10* are of unknown function, we gained most insight regarding interactions 3, 5 and 6 (Table 1). *ASTL* and *CYP11B2* were found to interact via two genes – *MEP1B* and *CYP2C9* (Fig. 2a). A functional partner of *EZR* was found to interact with MAP2K5 through other participants in the *MAPK* signaling pathway (Fig. 2b). Lastly, a member of the *IGF1R* protein complex was found to interact with *CAV3* (Fig. 2c).

**Table 1:** Results for QMDR association analysis for continuous BMI outcome

Rank	Model	SNP1	Chr:bp	Gene1	SNP2	Chr:bp	Gene2	Permuted *P*-Value	Bonferroni corrected *P*-value	Explicit epistasis *P*-value
1	rs17171686,rs1427463	rs17171686	7:40335451	*C7orf10*	rs1427463	17:59923044	*POLG2*	<0.00011	0.01	<0.001
2	rs12617233,rs1427463	rs12617233	2:58893502	*FLJ30838*	rs1427463	17:59923044	*POLG2*	<0.00012	0.01	0.012
3	rs749457,rs1799998	rs749457	2:96159671	*ASTL*	rs1799998	8:143996602	*CYP11B2*	<0.00026	0.03	<0.001
4	rs12617233,rs12210959	rs12617233	2:58893502	*FLJ30838*	rs12210959	6:6121143	*F13A1*	<0.00038	0.04	0.003
5	rs3102976,rs997295	rs3102976	6:159110007	*EZR*	rs997295	15:65803397	*MAP2K5*	<0.00046	0.05	<0.001
6	rs2268484,rs8038415	rs2268484	3:8748950	*CAV3*	rs8038415	15:97316957	*IGF1R*	<0.00046	0.05	0.009
7	rs12617233,rs822682	rs12617233	2:58893502	*FLJ30838*	rs822682	12:51798711	*SOAT2*	<0.00061	0.06	0.018

Seven signals reached a Bonferroni corrected *P*-value < 0.1. SNPs have been mapped to their corresponding genes using dbSNP (build 139) and SCANdb. SNP1 and SNP2 indicate the individual SNPs within a given SNP-SNP interaction model identified by QMDR. Chromosomal location of SNPs is noted in the following format - Chromosome: Base pair. *P*-values were calculated from a distribution built from 1000 permutations. *P*-values were also corrected using the Bonferroni method. Explicit epistasis *P*-values were calculated from a distribution built from 1000 permutations using the ‘explicit test of epistasis’

### Biofilter

Using the set of SNPs that emerged from the Biofilter procedure, QMDR analysis did not identify any significant SNP-SNP interaction models that were associated with BMI.

### Added variance in BMI explained

The reduced regression model including the main effect of the *FTO* index SNP and main effects of all SNPs within our identified interactions, had an adjusted R^2^ value of 0.008207. The full regression model including the main effects in the reduced model and the statistically significant SNP-SNP interactions identified, had an adjusted R^2^ value of 0.008932. Comparison of the two models showed a statistically significant increase in the variance explained (Likelihood ratio test *P*-value = 0.01).

### Replication analyses

After the use of an identical QMDR analysis procedure, we replicated the main effect filtered SNP-SNP interaction between rs749457 in *ASTL* and rs1799998 in *CYP11B2*. We also replicated 58 proxy SNP-SNP models representing this interaction in the eMERGE dataset at a permutation *P*-value threshold of 0.05 (Additional file 5: Table S4).

## Discussion

In this study, we analyzed the genetic and phenotypic information for a total of 15,737 individuals combined from five cohorts. SNPs were either filtered based on the strength of their independent effects or on the number of independent sources of biological knowledge supporting them. Filtered SNPs were then specifically tested for SNP-SNP interactions.

Historically, GWA studies have employed a linear modeling framework that tests single SNPs one at a time, for its association with a given phenotype. Hirschhorn et al. have shown that positive results from studies employing such an approach typically cannot be replicated across independent studies [39]. This has highlighted the need for embracing the complexity of a genotype-phenotype relationship by focusing on gene-gene interactions [40]. However, detecting gene-gene interactions in a GWAS presents a considerable computational and statistical challenge. Moore and Ritchie describe the need for designing new computational methods for detecting high-order non-linear interactions since traditional approaches such as logistic regression have limited power when modeling such interactions in high-dimensional data [41, 42]. They also stress upon the importance of filtering methods for the selection of SNPs to be included in an analysis. The exhaustive search of all possible combinations of thousands of SNPs is computationally very expensive. Our approach addresses both of these challenges. QMDR is a non-parametric method that does not assume any genetic model. Most importantly, QMDR greatly reduces the degrees of freedom required for modeling interactions. We also address the SNP-selection problem by applying two parallel filtering approaches, thereby effectively reducing our search space for detecting meaningful interactions.

We identified seven novel interactions that are highly associated with BMI. These seven interactions were also explicitly tested for the presence of epistasis. All the identified interactions exhibited an epistatic component. Moreover, we evaluated the increase in explained phenotypic variance by the identified SNP-SNP interactions. The index *FTO* SNP rs11642841 is in strong LD (r^2^ > 0.8) with the *FTO* SNP rs1558902 previously identified by Speliotes et al. to explain the largest proportion of the variance in BMI [1]. We found an 8.8 % increase in the variance in BMI explained by our identified SNP-SNP interactions, beyond what is explained by the main effects of the index *FTO* SNP and the SNPs within our interactions.

We found a significant association between rs749457 in *ASTL* and rs1799998 in *CYP11B2* related to BMI*.* This SNP-SNP interaction and 58 LD expanded models representing it were replicated in the eMERGE dataset. The variant rs1799998 has been associated with insulin resistance, diabetes, and metabolic syndrome in humans, but it has not been shown to have an independent association with BMI [43–45]. Little is known regarding the function of *ASTL* in humans, a specific protease that uses metals in catalytic processes [46]; however, there is moderate support connecting *ASTL* to a functional partner of *CYP11B2* (Fig. 2a). *ASTL* shares a strong sequence similarity and a common genetic ancestor with *MEP1A*. Both *MEP1A* and *MEP1B* are subunits of meprins and *MEP1B* shares a transcription factor binding site with and is part of the same gene expression signature as *CYP2C9*. Both *CYP2C9* and *CYP11B2* are functionally related by their roles in lipid metabolism [36]. *CYP11B2* is specifically involved in mineralocorticoid biosynthesis [47, 48]. Incidentally, the mineralocorticoid receptor has been shown to play an important role in the positive control of adipogenesis and thus, in the development of obesity [49].

In two interactions, rs1427463 in *POLG2* interacts with rs17171686 in *C7orf10* and rs12617233 in *FLJ30838* respectively. Although, little is known regarding the functions of *C7orf10* and *FLJ3083*, *POLG2’s* function may provide some insight into this interaction. *POLG2* is largely involved in metabolic pathways and the transcriptional activation of mitochondrial biogenesis [47, 48]. An increase in mitochondrial biogenesis has been shown to prevent the development of obesity in mice [50]. Conversely, mice with reduced expression of genes involved in mitochondrial respiration eventually develop obesity [51]. Consequently, the involvement of mitochondrial dynamics in obesity has gained a lot of support [52].

The SNP rs12617233 in *FLJ30838* also interacts with the SNPs rs12210959 in *F13A1*, and rs822682 in *SOAT2* respectively. *F13A1* encodes for the A subunit of the coagulation factor XIII and is involved in fibrin clot formation [53]. Several SNPs on this gene were found to be highly associated with BMI in a study utilizing gene expression data from monozygotic twins to deeply interrogate GWAS data [54]. Interestingly, the SNP identified in our study is independent of the *F13A1* signals identified by Naukkarinen et al. While several studies in obese individuals and rodent models of obesity have also reported increased levels of coagulation factors [55, 56], the exact mechanism of action is unknown. *SOAT2* is a major regulator of cholesterol metabolism and absorption in the small intestine and liver of mice on a high-cholesterol and high-fat diet [57] and impaired cholesterol absorption has been linked to high BMI and obesity [58, 59] through a yet unknown mechanism.

We also observed an interaction between rs997295 in *MAP2K5* and rs3102976 in *EZR* related to BMI. MAP2K5 is a part of the MAPK signaling pathway involved in growth factor stimulated cell proliferation. *EZR*, or ezrin, encodes a cytoplasmic peripheral membrane protein that links the plasma membrane and the actin cytoskeleton. Consequently, ezrin plays an important role in cell adhesion, migration, organization, and regulation of the actin cytoskeleton. Prior experimental evidence supports the physical and functional connection between these two genes (Fig. 2b) [36]. One can imagine the strong need for regulating the actin cytoskeleton during dynamic processes such as adipogenesis.

Lastly, we observed an interaction between rs2268484 in *CAV3* and rs8038415 in *IGF1R* related to BMI. *CAV3* encodes for a muscle-specific form of the caveolin family of proteins. Researchers have found that *CAV3*-knockout mice develop insulin resistance in their skeletal muscles [60] and that adenovirus-mediated gene transfer of *CAV3* increases glycogen synthesis in the liver as well as improves insulin signaling in diabetic obese mice [61]. *IGF1R* codes for the receptor of insulin-like growth factor 1, IGF1. IGF1 regulates pancreatic β-cell mass and thus plays a crucial role in insulin signaling. Hence, impaired *IGF1* signaling may alter insulin secretion by β-cells and negatively impact the hypothalamus – a region of the brain associated with food intake – ultimately causing weight gain [62]. Interestingly, a member of the *IGF1R* protein complex assembly (*RAS1*) is known to interact with a functional partner of *CAV3* in a number of processes such as signal transduction, endocytosis and focal adhesion (Fig. 2c) [36].

Four of the seven interactions that we identified include SNPs that have previously been identified as independent signals associated with BMI [27]. These SNPs are – rs12617233 in *FLJ30838* and rs997295 in *MAP2K5* – within interactions 2, 4, 5 and 7 (Table 1). *FLJ30838* is a long intergenic non-coding RNA (lincRNA) of unknown function. It was found to interact with rs1427463 in *POLG2*, rs12210959 in *F13A1*, and rs822682 in *SOAT2*. Incidentally, none of these other SNPs have been implicated in obesity before. The rs1427463 variant has been associated with height previously in an African ancestry population, which obviously factors into BMI calculations [63].

The use of the IBC array in this study highlights the strengths of this custom array in detecting potentially disease-causing loci that are also supported by a substantial amount of biological evidence. However, while the array has dense coverage in gene-centric regions, it only includes 2000 loci. This limitation of the array was highlighted by the inability of BioFilter to identify any statistically significant SNP-SNP models. The use of methods such as BioFilter may be more suited for larger GWAS datasets including more loci.

This study identifies gene-gene interactions that are potentially associated with obesity. Prior experimental evidence suggests the plausible biological relevance of several of the identified loci. However, we also identified a few loci of unknown function. Unfortunately, the inferences that can be drawn from our results are limited by a functional annotation bias – well-studied genes are assigned many annotations while understudied genes often lack annotations. One could speculate that the genes involved in these interactions are multi-functional, thereby connecting various biological processes and pathways. Future work focusing on network-based analyses can help elucidate the additional heritability of BMI that is explained at the biological pathway level. Ultimately, further biological validations will be necessary to determine whether the identified interactions influence the development of obesity. Finally, additional studies are required to better understand gene-environment interactions, to get a more complete understanding of the complex genetic architecture of obesity.

## Conclusions

Main effects analyses have explained little of the genetic heritability of obesity. The use of methods such as QMDR in conjunction with traditional statistical analyses can unravel this complex network by identifying gene-gene interactions that play key roles in the etiology of obesity. Our QMDR analysis of genotypic data from 5 study cohorts identified novel interactions between genetic variants that are highly associated with BMI. Future studies are necessary to verify the observed associations.

## Additional files

Additional file 1: Table S1. Information for cohorts providing individual level data. Information regarding the geographic location, and numbers of individuals included from each cohort. (PDF 40 kb) Additional file 2: Figure S1. Example of Cartesian product coding. An illustrative example of cartesian product coding used for interactions between SNPs within regression models to test non-additive nature of identified pairwise interactions. (PDF 46 kb) Additional file 3: Table S2. Information of eMERGE cohorts providing individual level data for replication analyses. Information regarding the geographic location, and numbers of individuals included from each cohort. (XLSX 39 kb) Additional file 4: Table S3. Details of LD expanded models. Number of LD-expanded (proxy) SNP-SNP models generated for each original discovered SNP-SNP model. (XLSX 34 kb) Additional file 5: Table S4. Results from QMDR association analysis of main effect filtered SNP-SNP models replicated in eMERGE dataset. Shown here are models that reached a permutation P-value < 0.05 in the replication dataset. SNPs have been mapped to their corresponding genes using dbSNP (build 139). SNP1 and SNP2 indicate the individual SNPs within a given SNP-SNP interaction model. P-values were calculated from a distribution built from 1000 permutations. (XLSX 53 kb) Additional file 6: Additional Acknowledgements. Acknowledgements and detailed descriptions of the five studies that provided the data for the analyses in this paper. (PDF 68 kb)

## Abbreviations

BMI: Body Mass Index
GWAS: Genome Wide Association Study
QMDR: Quantitative Multifactor Dimensionality Reduction
SNP: Single nucleotide polymorphism
MAF: Minor allele frequency
LD: Linkage disequilibrium
