# Supplementary material for: Identifying gene-gene interactions that are highly associated with Body Mass Index using Quantitative Multifactor Dimensionality Reduction (QMDR)
Source: BioData Min. 2015 Dec 14;8:41. doi: 10.1186/s13040-015-0074-0 (PMC4678717; doi:10.1186/s13040-015-0074-0)
Supplement: Additional file 1: Table S1. — Information for cohorts providing individual level data. Information regarding the geographic location, and numbers of individuals included from each cohort. (PDF 40 kb) [file 13040_2015_74_MOESM1_ESM.pdf]

Supplementary Table 1. Information for cohorts providing individual level data

Cohort No.	Cohort Name	Geographic Location	No. in Sample
1	ARIC	Washington County, MD; Forsyth County, NC; Jackson, MS; and Minneapolis, MN	9217
2	CARDIA	Birmingham, AL; Minneapolis, MN; Chicago, IL; and Oakland, CA	1350
4	CHS	Sacramento, CA; Hagerstown, MD; Winston-Salem, NC; and Pittsburgh, PA	3348
5	FHS	Framingham, MA	2484
6	MESA	New York, NY; Baltimore, MD; Chicago, IL; Los Angeles, CA; Twin Cities, MN; and Winston-Salem, NC	2287
		**Total Sample Size**	**18686**
