# Supplementary material for: Identifying gene-gene interactions that are highly associated with Body Mass Index using Quantitative Multifactor Dimensionality Reduction (QMDR)
Source: BioData Min. 2015 Dec 14;8:41. doi: 10.1186/s13040-015-0074-0 (PMC4678717; doi:10.1186/s13040-015-0074-0)
Supplement: Additional file 2: Figure S1. — Example of Cartesian product coding. An illustrative example of cartesian product coding used for interactions between SNPs within regression models to test non-additive nature of identified pairwise interactions. (PDF 46 kb) [file 13040_2015_74_MOESM2_ESM.pdf]

Supplementary Figure 1. Example of Cartesian product coding. An illustrative example of cartesian product coding used for interactions between SNPs within regression models to test non-additive nature of identified pairwise interactions

		**SNP1**		
		**aa (2)**	**Aa (1)**	**AA (0)**
**SNP2**	**tt (2)**	0	1	2
	**Tt (1)**	3	4	5
	**TT (0)**	6	7	8
