# Supplementary material for: Identifying gene-gene interactions that are highly associated with Body Mass Index using Quantitative Multifactor Dimensionality Reduction (QMDR)
Source: BioData Min. 2015 Dec 14;8:41. doi: 10.1186/s13040-015-0074-0 (PMC4678717; doi:10.1186/s13040-015-0074-0)
Supplement: Additional file 6: Additional Acknowledgements. — Acknowledgements and detailed descriptions of the five studies that provided the data for the analyses in this paper. (PDF 68 kb) [file 13040_2015_74_MOESM6_ESM.pdf]

Additional Acknowledgements

ARIC: The Atherosclerosis Risk in Communities Study is carried out as a collaborative study supported by National Heart, Lung, and Blood Institute contracts (HHSN268201100005C, HHSN268201100006C, HHSN268201100007C, HHSN268201100008C, HHSN268201100009C, HHSN268201100010C, HHSN268201100011C, and HHSN268201100012C), R01HL087641, R01HL59367 and R01HL086694; National Human Genome Research Institute contract U01HG004402; and National Institutes of Health contract HHSN268200625226C. The authors thank the staff and participants of the ARIC study for their important contributions. Infrastructure was partly supported by Grant Number UL1RR025005, a component of the National Institutes of Health and NIH Roadmap for Medical Research; **CARDIA**: Coronary Artery Risk in Young Adults: University of Alabama at Birmingham (N01-HC-48047, N01-HC-95095), University of Minnesota (N01-HC-48048), Northwestern University (N01-HC-48049), Kaiser Foundation Research Institute (N01-HC-48050), Tufts-New England Medical Center (N01-HC-45204), Wake Forest University (N01-HC-45205), Harbor-UCLA Research and Education Institute (N01-HC-05187), University of California, Irvine (N01-HC-45134, N01-HC-95100); **CHS**: This research was supported by contracts HHSN268201200036C, N01HC85239, N01 HC55222, N01HC85079, N01HC85080, N01HC85081, N01HC85082, N01HC85083, N01HC85086, and grant HL080295 from the National Heart, Lung, and Blood Institute (NHLBI), with additional contribution from the National Institute of Neurological Disorders and Stroke (NINDS). Additional support was provided by AG023629 from the National Institute on Aging (NIA). A full list of principal CHS investigators and institutions can be found at http://www.chs-nhlbi.org/pubs/PubAcknowGuidelines.htm; **FHS**: The Framingham Heart Study began in 1948 with the recruitment of an original cohort of 5,209 men and women (mean age 44 years; 55 percent women). In 1971 a second generation of study participants was enrolled; this cohort consisted of 5,124 children and spouses of children of the original cohort. The mean age of the offspring cohort was 37 years; 52 percent were women. A third generation cohort of 4,095 children of offspring cohort participants (mean age 40 years; 53 percent women) was enrolled beginning in 2002. At each clinic visit, a medical history was obtained with a focus on cardiovascular content, and participants underwent a physical examination including measurement of height and weight from which BMI was calculated; **MESA**: The Multi-Ethnic Study of Atherosclerosis Study (MESA) is a multicenter prospective cohort study initiated to study the development of subclinical cardiovascular disease. A total of 6814 women and men between the age of 45 and 84 year were recruited for the first examination between 2000 and 2002. Participants were recruited in six US cities (Baltimore, MD; Chicago, IL; Forsyth County, NC; Los Angeles County, CA; Northern Manhattan, NY; and St. Paul, MN). This study was approved by the institutional review boards of each study site, and written informed consent was obtained from all participants. This cohort was genotyped as part of the National Heart Lung and Blood Institute's (NHLBI) Candidate Gene Association Resource (CARe) (Musunuru, K., Lettre, G., Young, T., Farlow, D.N., Pirruccello, J.P., Ejebe, K.G., Keating, B.J., Yang, Q., Chen, M.H., Lapchyk, N. et al. Candidate gene association resource (CARe): design, methods, and proof of concept. Circ. Cardiovasc. Genet, 3, 267-275.).
